# The promise of resurrection plants in enhancing crop tolerance to water scarcity

**DOI:** 10.1098/rstb.2024.0231

**Published:** 2025-05-29

**Authors:** Shumayla, Gerardo Alejo-Jacuinde, Pablo Silva-Villatoro, Chidinma Lois Nwoko, Melvin J. Oliver, Luis Herrera-Estrella

**Affiliations:** ^1^ Plant and Soil Science, Texas Tech University, Lubbock, TX, USA; ^2^ Division of Plant Science and Technology, University of Missouri, Columbia, MO, USA; ^3^ Unidad de Genómica Avanzada/LANGEBIO, Cinvestav Irapuato Unit, Irapuato, Guanajuato, Mexico

**Keywords:** drought, climate change, vegetative desiccation tolerance, resurrection plants, crop engineering, crop sustainability

## Abstract

Climate change affects the agricultural sector by modifying precipitation patterns, increasing extreme weather events, and geographically shifting agriculturally viable areas. These climate alterations substantially impact plant resilience to abiotic stress and, consequently, agricultural productivity. A better understanding of plant adaptations to tolerate extreme environmental conditions could pave the way for future advances in agricultural sustainability. One such adaptation is vegetative desiccation tolerance (VDT), which enables some species, known as ‘resurrection plants’, to undergo almost complete drying without losing viability. The current review discusses how incorporating different molecular and biochemical mechanisms underlying VDT into crops might expand the time during which crops can continue growing under limiting water conditions and perhaps broaden the range of survivable negative water potentials that a crop can endure under drought stress. Such possibilities could alleviate the detrimental consequences of low water availability to crops. Understanding how plants survive extreme dehydration has the potential to enlighten new strategies to improve the climate resiliency of crops, thereby positively impacting worldwide food security and sustainability.

This article is part of the theme issue ‘Crops under stress: can we mitigate the impacts of climate change on agriculture and launch the ‘Resilience Revolution’?’.

## 1. Introduction

Drought is the greatest threat to worldwide agriculture and increased and extended droughts are predicted worldwide due to global warming. This threatens global food security and represents an existential issue for humankind. Drought-induced soil moisture deficits trigger a cascade of severe environmental consequences, including soil erosion, heightened salinity, reduced agricultural productivity, altered ecosystems, and increased wildfire risk. This global phenomenon significantly impacts plant growth and productivity [1,2]. Surprisingly, 60–80% of the world’s arable land is arid or semiarid and the United Nations World Water Development Report and the Intergovernmental Panel on Climate Change predict a 20–30% increase in water demand by 2050 [2,3]. Water scarcity and drought affect nearly half of the world’s cereal crops, occupying almost 70% of the total agricultural land.

**Figure 1.**
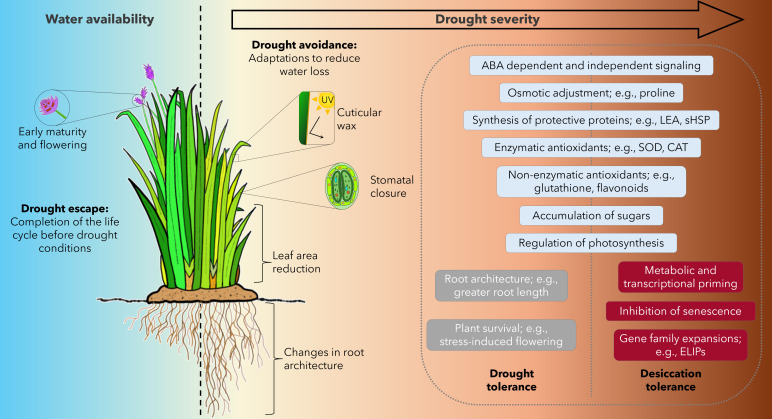
Plant adaptation and mechanisms to cope with water stress. A schematic representation showing varying degrees of water stress (left to right arrow indicates water stress severity), and the different adaptations and protection mechanisms observed in plants under drought conditions. ABA, abscisic acid; CAT, catalase; ELIPs, early light inducible proteins; LEA, late embryogenesis abundant; sHSP, small heat shock proteins; SOD, superoxide dismutase.

Due to their sessile nature, plants are frequently exposed to environmental stress, including water deficits. Low water availability significantly impacts crop performance by altering physiological and biochemical processes, impairing nutrient uptake, decreasing photosynthetic efficiency, and reducing biomass accumulation [4]. Water stress particularly affects reproductive development and seed set, limiting pollination and grain filling, ultimately reducing the final yield [5]. For instance, pearl millet yields can decline to between 828 and 1136 kg ha^−1^ under drought conditions, compared to 3123–3942 kg ha^−1^ in adequately watered environments [6]. Additionally, maize and wheat also experience yield losses due to water-stress-related stomatal closure, impaired nutrient transport and oxidative damage [7]. Throughout evolution, plants have acquired various mechanisms and adaptations to cope with water stress (figure 1), including enhanced water-holding capacity by regulating stomata opening and limiting shoot growth, maintenance of leaf morphological structure, improved root growth for water absorption, accumulation of osmoprotectant metabolites, and the regulation of reactive oxygen species (ROS) scavenging systems that reduce oxidative damage [8–10]. Certain cash crops are adapted to mild-to-moderate water stress, however, most are still vulnerable to extreme or long periods of drought because of their limited capacity to survive and recover from severe water loss.

The most successful adaptation to extreme water loss is vegetative desiccation tolerance (VDT). It is a remarkable adaptation that allows certain plants to endure extreme cellular dehydration and recover upon rehydration [11]. Desiccation-tolerant plants, also known as ‘resurrection plants’, can withstand desiccation of their vegetative tissues and recover from a state with a water potential below −100  MPa where insufficient water is available to support biological activity [12]. VDT was thought to have evolved 500 million years ago and was likely essential in the shift of primitive plants from aquatic to terrestrial environments [13]. Early land plants presumably had to withstand periods of dryness, and as modern-day algae and bryophytes, their cellular water potential would equilibrate to that of the surrounding air once surface water was absent; thus, in dry air, VDT was critical for survival [14]. Evolutionary analyses indicated that VDT was lost during the evolution of tracheophytes (vascular plants) but re-evolved in some genera within several plant lineages, including lycophytes, ferns, and angiosperms [12,15].

Over the past two decades, ‘omics’ approaches have been extensively utilized to better understand the biochemical and molecular mechanisms that resurrection species activate to tolerate the desiccation (and rehydration) process. The mechanisms underlying VDT involve cellular protection and recovery processes [12]. During dehydration, desiccation-tolerant plants accumulate sugars (e.g., raffinose and sucrose) and nitrogen-based osmoprotectants, stabilizing cellular structures. Antioxidant enzymes, late embryogenesis abundant (LEA) proteins, and additional protective proteins also accumulate to cope with desiccation [14,16,17]. Resurrection angiosperms also suppress senescence, allowing the plants to use their resources for protection and recovery [12,18]. Although significant progress has been made in understanding VDT through omics approaches, several questions remain open, including the exact nature of the regulatory circuits controlling this trait and its key genomic signatures.

Resurrection plants have tremendous potential for providing novel tools and strategies for improving water stress tolerance in crops to ensure food security in a world where water is becoming progressively scarce. As the world is increasingly facing challenges with climate change, the idea of engineering crops that are better adapted for more frequent and extreme drought events becomes more appealing. However, some crucial concerns still need to be addressed: What are the critical adaptations and genetic elements enabling resurrection plants to withstand extreme dehydration? How do the resurrection plants maintain their cellular integrity? Most importantly, can these traits be transferred to crops to improve tolerance while maintaining high yield?

This review provides an overview of the distinct adaptations plants employ in drought conditions and the critical differences between drought and desiccation tolerance (DT). We highlight the problems faced during drought and how they could be tackled using knowledge from the study of resurrection plants. The review also discusses the latest advancements in understanding VDT, emphasizing the molecular, biochemical and physiological adaptations that enable resurrection plants to survive desiccation. Furthermore, we highlight the research done so far and the unexplored areas that need attention in future studies.

## 2. Drought stress adaptations in plants

In plants, drought stress causes osmotic damage, oxidative stress, and metabolic dysfunction, hampering plant growth, inducing leaf, flower, and fruit abscission, and ultimately leading to plant death [19–21]. However, the impact on the plant depends on the severity of the water deficit, usually assessed by declining leaf water potentials, that develops within the plant during a drought event. By its very nature, the stress that the plant experiences as the soil dries during a drought progresses along a gradient from mild to moderate to severe stress levels, ultimately culminating in plant death. Specifying at which water potential a plant enters mild stress is complex, although any decline in leaf water potential from a fully hydrated state would signal the potential for stress. At what water potential a plant transitions into moderate or severe stress is also difficult to determine as it varies significantly from species to species. For most crop plants, the maximum range of survivable leaf water potentials is relatively narrow, from fully hydrated to −5.0 MPa [12]. However, plants generally exhibit distinguishable characteristics at each stage of the stress gradient. Mild drought stress is characterized by a slight decline in leaf water potential, resulting in a subtle but significant decrease in photosynthesis and growth. Stomatal closure is incomplete, allowing plants to sustain metabolic activity and turgor via osmotic adjustment and antioxidant responses [22]. After mild drought stress, plants can recover when water becomes available. However, mild drought stress has a detrimental effect, reducing crop biomass and yield. Moderate drought stress results in a substantial decline in growth and photosynthesis because of complete stomatal closure to eliminate water loss via transpiration, although turgor is still maintained. Chlorophyll breakdown and increased oxidative stress stimulate the synthesis of protective proteins and elevate antioxidant enzyme activity [23]. Enhancing tolerance to moderate drought is complex, requiring genetic selection for traits such as deeper root systems and improved water-use efficiency [24]. Severe drought stress results in loss of turgor, cessation of growth, stomatal closure, and the uncoupling of photosynthesis. Extensive exposure to severe drought results in irreversible tissue damage, senescence, and death in considerable measure because of oxidative damage [25].

When plants encounter drought during critical growth phases, stress-induced damage can significantly reduce crop yields [26–28]. For instance, the most sensitive growth phase in maize to drought stress is during flowering, including ten days before and after pollen shed, directly affecting kernel set and yield [29]. Water scarcity can impact plants at any stage of their life cycle, and its effects may vary among different crop species. Some studies have shown that wheat is more susceptible to water shortages during leaf development compared to maize [30] and sunflower [31]. In leguminous plants, water deficits adversely affect root nodule formation, resulting in reductions in nodule size, biomass, and nitrogenase activity. Plants employ various mechanisms to cope with drought stress. These include developmental, cellular, and morphological adaptations such as shorter anthesis-silking intervals, stomatal regulation to control transpiration and minimize water loss [32–34], altering leaf angles to reduce sunlight exposure [35], increased root elongation to access deeper water reserves [36], and maintaining low water potentials in tissues for efficient water usage [37]. Plants also activate physiological responses to promote osmotic adjustment to slow water loss, retain cell structure, protect photosynthetic apparatus through photoprotective pigments and proteins, reduce cytoplasmic viscosity, and increase tissue succulence [38]. Additionally, plants experiencing drought accumulate protective compounds to safeguard cellular macromolecules [39].

Plants have evolved into three broad phenotypic categories, defined by how they cope with drought events (figure 1): drought escape, avoidance, and tolerance [19,36,40–46]. Drought escape centres on the plant completing its entire life cycle swiftly during periods of optimal water availability, effectively avoiding drought and circumventing the adversities caused by water stress. This is achieved by hastening reproductive processes, including flowering and seed production, so they are completed before the onset of drought [43,47,48]. Drought avoidance in plants involves mechanisms to store water (e.g., succulence) or to minimize water loss by reducing leaf area and regulating stomatal apertures to decrease transpiration and conserve water [49,50]. However, drought tolerance enables the plant to accommodate the stressful effects of dehydration and recover once the stress is relieved. Drought avoidance and drought tolerance are the primary mechanisms by which plants can achieve drought resistance (resistance to the rigours of a water stress event) [51].

## 3. Water stress responses: from drought to desiccation biology

**Figure 2.**
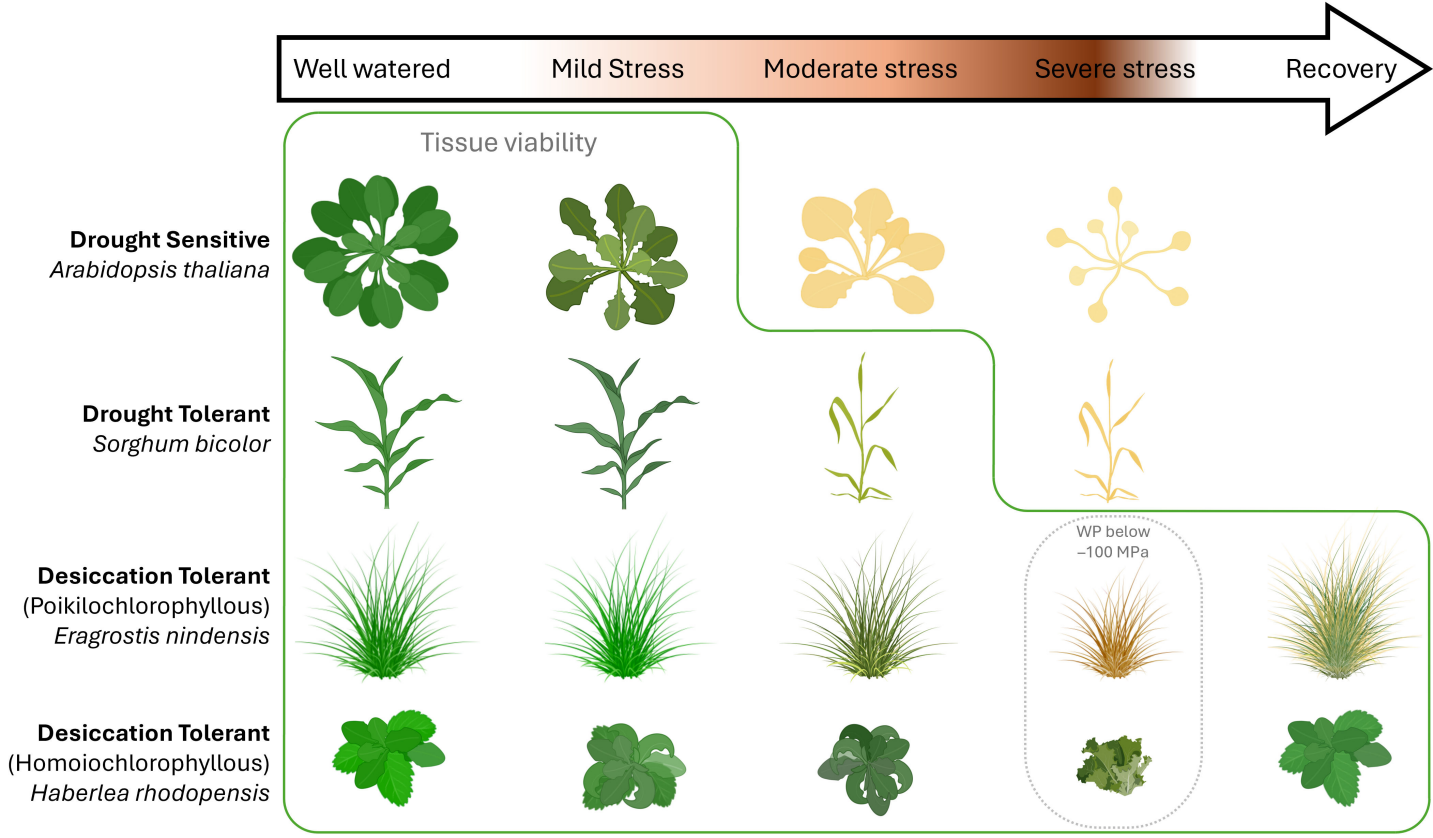
Variations in plant survival across a progressive water stress gradient. This stress gradient is depicted with arrow colour intensity representing stress severity (mild, moderate and severe stress), where darker shades indicate more severe stress. Examples of plant species with varying degrees of water stress tolerance. Green solid outline indicates viable plants that withstand drought conditions. WP, water potential.

Although sometimes used interchangeably, drought tolerance and desiccation tolerance represent distinct adaptations to water loss. The former is an adaptation to limit water loss from the plant, and the latter is an adaptation that enables an organism or tissue to endure and survive extreme dehydration at the cellular level [52] (figure 2).

Under water stress conditions, drought-tolerant plants maintain sufficient cellular water to support metabolic activity and exhibit several responses and mechanisms to avoid reaching a critical water deficit that would cause irreversible damage [43]. There is usually a higher accumulation of abscisic acid (ABA), a central plant hormone that responds to water deficit stress, which triggers the expression of drought-responsive genes [25,53,54] along with the accumulation of osmolytes like proline, glycine betaine, and sugars to maintain cell turgor and prevent shrinkage [53,55]. Some protective proteins, such as dehydrins and LEA, are produced to stabilize cellular components during drought stress [56,57]. Drought-tolerant plants may also have morphological adaptations, such as smaller, thicker leaves, waxy cuticles, and altered stomatal density to minimize water loss and optimize gas exchange [53,58]. These strategies enable plants to survive in arid and semi-arid regions. Drought-tolerant crop plants can tolerate leaf water potentials that approach the maximum stress level of approximately −5 MPa, a relatively narrow tolerance range compared to the demands imposed by extreme drought conditions [59]. This limited tolerance results in significant crop losses during long periods of water deficit stress, as crops cannot maintain cellular turgor and critical metabolic functions as the stress becomes more severe when the soil dries to water potentials that exceed the ability of the plants to retain water [22]. The ability of plants to maintain viability at lower soil water potentials is a crucial target for enhancing drought resilience in crops, particularly in agricultural systems such as subsistence farming, to help withstand and survive prolonged and severe drought conditions [23].

Desiccation tolerance (DT) is a survival strategy that allows organisms to endure drying to equilibrium with the water potential of the surrounding air with minimal cellular damage and recover when water is reintroduced [60–62]. For plants, VDT has been defined as the ability to survive drying to a water potential equal to or below −100 MPa (equivalent to equilibration with air at 50% relative humidity at 20°C) [12]. This phenomenon is common in reproductive structures such as seeds, pollen, and spores but only occurs in the vegetative tissues in a relatively few species, including some bryophytes, lycophytes, ferns, and angiosperms [12]. Recent studies have found more than 600 terrestrial plants that can tolerate desiccation, including members of 68 bryophyte families, 10 fern and fern allied families, and 10 angiosperm families [63–65]. The phenomenon of VDT is exemplified by bryophytes such as *Syntrichia caninervis* and *Syntrichia ruralis*, ferns like *Polypodium polypodioides*, fern allies such as *Selaginella lepidophylla*, and angiosperms including *Craterostigma plantagineum* and *Xerophyta schlechteri* (previously identified as *X. viscosa*) [56,66,67]. All these plants can lose almost all their cellular water and remain quiescent for extended periods before resuming metabolic activity upon rehydration [13].

In summary, desiccation and drought tolerance differ significantly in terms of the stress level the plants can endure. While drought and DT involve coping with water scarcity, DT is a more extreme survival trait. Drought tolerance can be seen as a continuum, where plants have evolved mechanisms to avoid or withstand moderate water deficits. On the other hand, DT involves surviving extreme cellular dehydration and is often observed in habitats where water availability is highly variable or drought events are prolonged [53]. It represents a unique adaptation that goes beyond the typical mechanisms of drought tolerance seen in most plants.

## 4. Adaptations of resurrection plants

**Figure 3.**
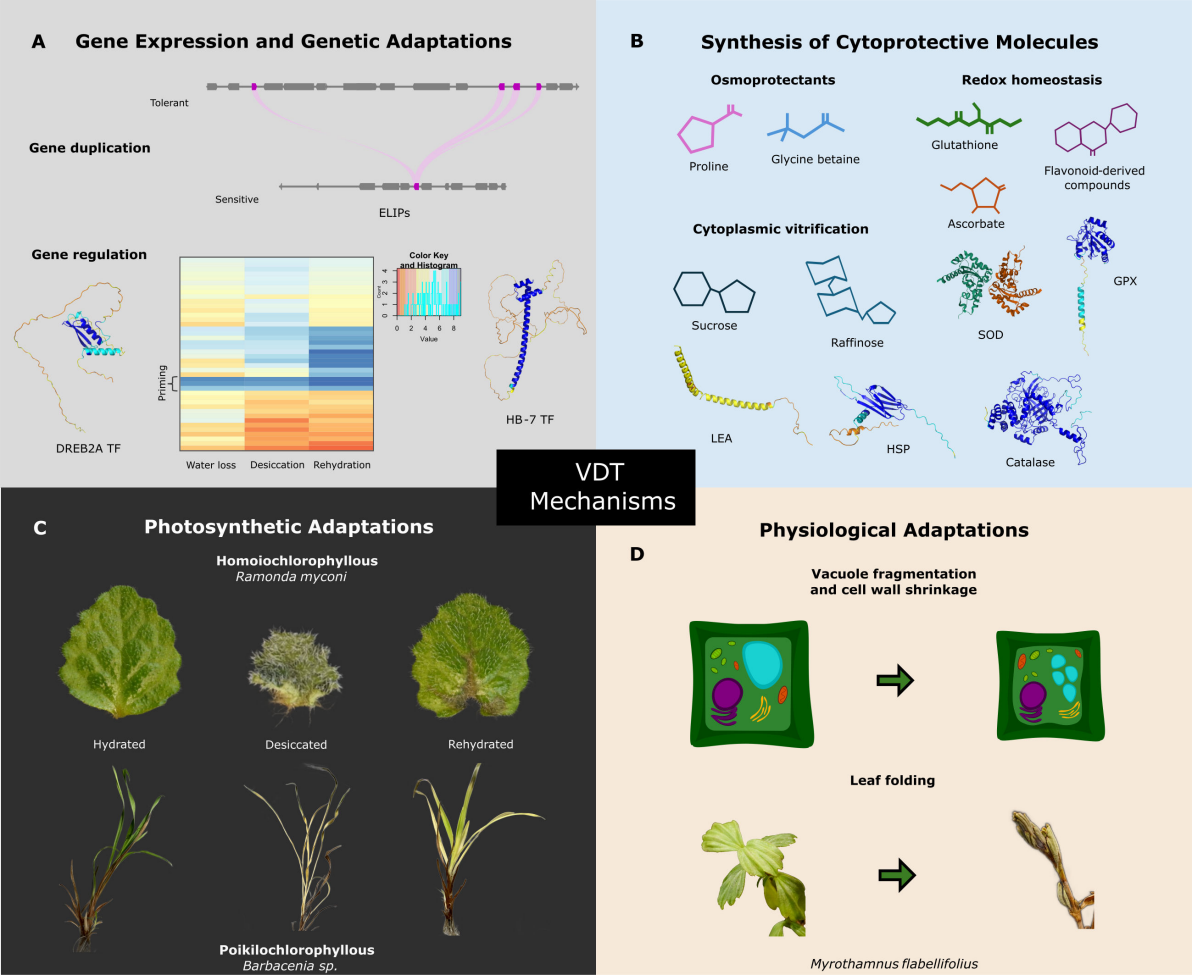
Overview of vegetative desiccation tolerance (VDT) mechanisms. This figure illustrates key mechanisms associated with VDT across various biological adaptations. (A) Genomic features associated with VDT are represented with the gene duplication of the early light-induced proteins (ELIPs) genes family. Gene regulation is shown through transcription factors (TFs), including DREB2A and HB-7. Hypothetical transcript abundance patterns of protection-related genes (heatmap) suggesting dynamic gene activity adjustments during desiccation. (B) Desiccation-tolerant plants accumulate high levels of compounds that help to maintain osmotic balance and stabilize cellular structures (e.g., proline and glycine betaine). Molecules like glutathione, ascorbate, raffinose, and flavonoid-derived compounds play key roles in managing oxidative stress. Additionally, enzymatic antioxidant response includes glutathione peroxidase (GPX), superoxide dismutase (SOD), and catalase. Carbohydrates such as sucrose in combination with late embryogenesis abundant (LEA) proteins contribute to cytoplasmic vitrification, a process where the cytoplasm transitions into a glass-like state to avoid damage during desiccation. (C) Resurrection plants exhibit two types of photosynthetic strategies: homoiochlorophyllous (species that retain the photosynthetic apparatus during desiccation) or poikilochlorophyllous (species that dismantle their photosynthetic apparatus during dehydration and resynthesize it upon rehydration). Images show its hydrated, desiccated, and rehydrated states. (D) Representation of physiological adaptations such as vacuole fragmentation and cell wall shrinkage. Hydro-responsive leaf folding in the species *Myrothamnus flabellifolius*, a physical adaptation that protects internal tissues from light and heat stress.

For a plant cell to survive desiccation, it requires the action of multiple pathways and genes (figure 3), whose products interact to finely tune subcellular modifications and, consequently, promote tissue survival. Molecular insights are crucial for understanding the mechanisms underlying DT and identifying possible targets for improving drought tolerance in crop plants. These insights are often obtained through transcriptomics, proteomics, and metabolomics, which have identified key transcripts, proteins, and metabolites involved in plant responses to various levels of water loss and rehydration. In conjunction with bioinformatic analysis, biochemical and physiological studies are necessary to validate the functional roles of these molecules as protective or regulatory agents. This integrated approach can uncover new components to enhance drought tolerance that could be implemented through targeted breeding or genetic modification into crops.

It is important to note that desiccation-tolerant plants activate several responses observed during dehydration stress in desiccation-sensitive plants and some of the mechanisms that confer DT in seeds. Although desiccation and dehydration responses in tolerant and sensitive species share some biochemical and molecular similarities, there are distinct differences. This is best illustrated through sister group contrast studies, which compare closely related species that differ in their tolerance to desiccation. Sister group studies have demonstrated that some desiccation-tolerant species are poised or primed metabolically to tolerate desiccation. Some desiccation-tolerant *Selaginella* species accumulate high levels of sucrose and sugar alcohols, and an elevated antioxidant capacity under non-stressed conditions compared to their desiccation-sensitive sister species [68,69]. A recent sister group study shows that the desiccation-tolerant *Selaginella sellowii* is transcriptionally and metabolically primed for VDT [70]. Specifically, the constitutive expression of flavonoid-related genes and the high abundance of flavonoid compounds under hydrated conditions represent a key difference between tolerant and sensitive *Selaginella* species. Similarly, the desiccation-tolerant grass *Sporobolus stapfianus* maintains high levels of osmolytes and nitrogenous osmoprotectant compounds in the hydrated state, which are not observed in the hydrated sensitive sister species *Sporobolus pyramidalis* [71,72]. It has been suggested that the accumulation of these osmoprotective compounds allows the plants to slow water loss such that the programmes that establish VDT can be activated and established before the cells reach a water content that is non-conducive to metabolic activity [73]. In the *Sporobolus* metabolomic contrast, *S. stapfianus* responded rapidly during the initial stages of dehydration whereas *S. pyramidalis*, even though wilting (loss of turgor), failed to activate the stress protection mechanism and likely did not until it was too late to prevent damage [71]. Although the transcriptomic response was extensive for both species in the early stages of dehydration, the types of transcripts that exhibited differential accumulation were extensively different, with only a limited overlap [74], indicating that the water stress response of the sensitive *S. pyramidalis* was very different from that of the tolerant *S. stapfianus*. Similar differences were observed comparing desiccation-tolerant and desiccation-sensitive dicot species within the Linderniaceae. Transcriptomic comparisons between the tolerant *Lindernia brevidens* and the sensitive species *Lindernia subracemosa* revealed the activation of very different gene networks in response to dehydration and again, in this contrast, the response of the sensitive species occurred only when cellular damage was imminent [75].

### (a) Biochemical adaptations

Desiccation-tolerant plants activate several mechanisms to stabilize cellular structures and prevent excessive damage during water loss and subsequent rehydration [12,56,72]. Among the key molecular responses is the accumulation of LEA proteins, which are considered an essential component of VDT. These intrinsically disordered proteins are thought to be molecular chaperones that prevent protein aggregation and buffer cell membranes with a hydration shell [76]. Heat shock protein (HSP) genes are also highly induced during desiccation and are critical for maintaining protein structure under stress [76]. In addition to protein stabilization, resurrection plants have evolved carbohydrate-based mechanisms to enhance dehydration tolerance. In desiccation-tolerant plants, water loss promotes the accumulation of non-reducing sugars, especially sucrose and raffinose, which accumulate at high levels, replacing water molecules in cellular structures [77]. These sugars, along with LEA proteins, are also involved in cytoplasmic vitrification, a process that prevents crystallization of the cellular contents and thereby preserves cellular integrity during desiccation [78–80]. All these biochemical adjustments are essential for preserving cellular constituents during extensive drying and in the dry state, ensuring their functionality upon subsequent rehydration [81] (figure 3).

Dehydration-induced oxidative stress, primarily in the form of the accumulation of ROS, is countered by robust antioxidant defence systems in resurrection plants. These defences involve increased expression of enzymes like superoxide dismutases (SOD), catalases (CAT), and peroxidases, which scavenge ROS, thereby preventing damage at the cellular level [82]. The production of antioxidants such as ascorbate, glutathione, and raffinose act in conjunction with enzymatic antioxidants to maintain redox homeostasis during desiccation [76]. Besides the well-characterized enzymatic and non-enzymatic antioxidants, secondary metabolites such as flavonoid-derived compounds have also been proposed as essential for maintaining redox homeostasis in cellular organelles during drying [83].

Resurrection plants employ other biochemical mechanisms common to drought tolerance to survive desiccation, most notably the synthesis and accumulation of osmoprotectant compounds such as proline, glycine betaine, and polyols. These osmoprotectants are critical in adjusting cellular osmotic balance and protecting the organelles from membrane disruption [84]. During desiccation stress, metabolic flux undergoes a significant modification in resurrection species. A good example of this, as mentioned earlier, is an increase in carbon flux to the accumulation of raffinose family oligosaccharides (RFOs) during desiccation, which, along with their capacity as antioxidants, act as osmoprotectants and have various structural and chemical functions during rehydration. Activity-based protein profiling methods have identified their role in cell recovery [85]. Additionally, the induction of non-circadian respiratory pathways during dehydration is crucial for downregulating ROS generation, thereby reducing oxidative damage during periods of low water availability [86].

### (b) Molecular adaptations

Genes responsible for VDT are known to be tightly regulated at the molecular level, controlling a wide range of processes and pathways that mediate responses to water loss in these plants (figure 3). Transcriptomic analyses of resurrection species have provided insights into the changes in transcript abundance involved in VDT [87–93]. These studies have identified several transcription factor (TF) families that appear critical in mediating the VDT response. Among the most prominent TF families are the WRKY, dehydration-responsive element-binding protein (DREB), myeloblastosis (MYB), and homeodomain leucine zipper (HD-Zip) families. However, only a few studies have validated the direct participation of some TFs in VDT. WRKY TFs are important for mediating stress responses in plants, and the *BhWRKY1* gene was demonstrated to regulate galactinol synthase, an essential step in the synthesis of RFOs, in the desiccation-tolerant dicot *Dorcoceras hygrometricum* (previously identified as *Boea hygrometrica*) in an ABA-dependent manner [85,94]. The complexity of transcriptional regulation in VDT is emphasized in the dual role of the TF CpMYB10 in *C. plantagineum*. Under non-stressed conditions, this MYB TF acts as a repressor of not only its own expression but also of several stress-related genes. However, upon dehydration, it becomes an activator, likely via the activity of a protein kinase, to activate the expression of cellular protection-related genes like RD29A and LEA proteins [95]. This transcriptional flexibility underscores the intricacies of adaptive mechanisms employed in resurrection plants, where TFs finely tune gene expression in response to fluctuating water availability [96].

Transcriptional regulation during VDT is also dynamic, requiring the coordinated expression of protective genes at specific stages during dehydration. This is exemplified by the activity of TFs of the HD-Zip family. In *C. plantagineum*, the HD-Zip members CpHB-1, CpHB-2, CpHB-6 and CpHB-7 are upregulated in response to dehydration [97]. However, CpHB-6 and CpHB-7 participate only during early dehydration, and their transcript abundance decreases during prolonged dehydration. CpHB-2, CpHB-6 and CpHB-7 are induced by both dehydration and ABA, but CpHB-1 is only induced by dehydration, emphasizing the involvement of both ABA-dependent and independent signalling mechanisms [98], highlighting the multifaceted regulatory networks that underpin VDT [99]. While members of the HD-Zip TF family access ABA signalling to manage desiccation responses, the DREB TFs take a more direct route to impact DT by binding to dehydration-responsive *cis*-elements (DREs) in the promoters of dehydration-inducible genes [100]. For example, the DREB transcription factor BaDBL1 from the desiccation-tolerant moss *Bryum argenteum* is important for regulating lignin biosynthesis, particularly under osmotic stress conditions. Overexpression of BaDBL1 in transgenic *Arabidopsis* lines, when exposed to osmotic stress, increased lignin content and transcript abundance for lignin-biosynthesis genes, such as Phe ammonia-lyase (PAL), 4-coumaroyl CoA-ligase (4 CL), cinnamyol alcohol dehydrogenase (CAD), and cinnamoyl CoA reductase (CCR) to higher levels than observed in wild-type plants under the same stress. This indicated that BaDBL1 enhances lignin production as part of the plant’s response to dehydration. This modulation of the phenylpropanoid pathway may strengthen the cell wall, improving plant resilience to environmental stressors [101]. Other studies have also identified NAC (NAM, ATAF, and CUC) TFs as key regulators of stress responses, contributing to the regulation of genes involved in cell wall remodelling and protein stabilization during dehydration [102]. Overall, this diversity in the TF families participating in VDT underscores the complexity of the regulatory pathways that evolved in resurrection plants to desiccation.

### (c) Physiological adaptations

The physiological adaptations required for resurrection plants to recover from desiccation are diverse and complex, involving marked morphological alterations, cellular water relations, and photosynthetic activity [86,103] (figure 3). One of the most remarkable adaptations of some resurrection plants is their ability to change shape during dehydration. Leaf folding or rolling occurs in various species to reduce the surface area exposed to sunlight, thus slowing water loss and reducing ROS production from the uncoupling of photosynthesis during drying [67]. In *S. lepidophylla*, this curling is driven by differential tissue composition, particularly by variations in lignification and cell wall properties along the stem, and results in a significant reduction in the exposed surface area during dehydration [104]. Such remarkable morphological changes are thought to be primarily related to cell wall modifications, including composition and architecture, as these structures must become highly flexible to accommodate such drastic alterations in shape. Evidence suggests that these modifications involve changes in expansins, which make the cell walls more flexible, allowing for reversible deformations during desiccation [67,105]. Along with cell wall modifications, changes in lipid composition are also thought to be required to maintain cellular integrity during desiccation. An increase in unsaturated fatty acids associated with an increase in membrane fluidity and stability has been reported in some resurrection species [106]. Lipid modifications also prevent membrane phase transitions that may cause membrane damage and leakage during dehydration and rehydration [86]. Extreme cellular water loss leads to mechanical stress resulting from significantly reduced cellular volume. Some resurrection plants exhibit mechanisms that mitigate such mechanical stress, including vacuole fragmentation that minimizes the risk of vacuole membrane rupture and the associated cellular damage that would occur from the release of the vacuolar contents into the cytoplasm as cells shrink [107]. Vacuole fragmentation is a mechanism by which this integral cellular component is maintained throughout a dehydration–rehydration event.

Resurrection plants can be classified into homoiochlorophyllous and poikilochlorophyllous depending on whether they maintain or dismantle their photosynthetic apparatus during dehydration. Homoiochlorophyllous plants, such as *C. plantagineum*, maintain chlorophyll and thylakoid structures during desiccation, enabling them to resume photosynthesis rapidly upon rehydration. This retention has been proposed to be facilitated by the elevated expression of early light-inducible proteins (ELIPs), which protect the photosynthetic apparatus from damage during dehydration [84,108]. Retaining chloroplasts and chlorophyll, however, makes them susceptible to ROS production resulting from the uncoupling of photosynthesis during drying (as mentioned previously), and therefore, homoiochlorophyllous species evolved very efficient ROS scavenging mechanisms. In contrast, poikilochlorophyllous resurrection plants, such as *X. schlechteri*, dismantle their photosynthetic apparatus during drying, thus eliminating ROS generation via the electron transport pathway of photosynthesis and the associated oxidative damage during desiccation [107]. Given these different protective mechanisms, the regulation of photosynthesis in resurrection plants is of particular interest [76].

Expansion of the ELIP gene family through tandem duplications represents a molecular signature for VDT in the genomes of resurrection plants [108]. The transcripts for these proteins accumulate significantly during dehydration, and these proteins are thought to protect against photooxidative damage by binding free chlorophyll and stabilizing photosynthetic complexes [109]. The expansion of this gene family supports the hypothesis that VDT in land plants may have evolved through the duplication of stress-related genes, leading to multiple paralogs across resurrection species [108].

## 5. Harnessing the resilience of resurrection plants to enhance crop drought tolerance

As discussed earlier, severe water deficits lead to plants no longer maintaining turgor, the turgor loss point (TLP) or the wilting point [110], a central aspect of drought tolerance. Plants whose water status is above the TLP can maintain growth, but once plants reach the TLP and turgor is lost, growth is not possible, and cellular mechanisms to limit damage are employed. As the stress progresses and water potential continues to decline, there comes a point when damage limitation efforts fail, and the plant dies. The water potentials at which these events occur define the drought tolerance of a particular species. The TLP varies considerably from species to species, but the lethal water potential for most crops is between −3 and −5 MPa, with drought-tolerant lines at the lower end of the range. Extending the time above the TLP (or lowering the TLP) has been proposed as a strategy to boost drought tolerance [111], presumably to lower water potentials, but this requires a deeper understanding of the physiological, biochemical, and molecular mechanisms involved in plant tolerance and stress responses to severe water loss [86]. Nevertheless, lowering the TLP and thus maintaining the ability of the plant to continue growth is a target that can impact crop yields in the high-throughput production agriculture practiced in developed parts of the world. Lowering the water potential at which water deficit becomes lethal is a potential target for crop improvement. While this approach might expand the range of soil moistures (more arid regions) that one could plant a crop, it would do little to improve or maintain yields. Still, it could significantly impact subsistence agriculture, where total crop failure is unacceptable. In the above sections, we have discussed the various adaptations desiccation-tolerant plants employ and the potential components and processes that could be targeted for enhanced drought tolerance of crop plants. Genome engineering techniques could be utilized to lengthen the time to reach the TLP, lower the water potential at which it occurs, and expand the water potential survivable range to below −5 MPa [23].

Although some of the molecular and biochemical responses to dehydration appear to be common to drought and DT, the critical differences between these adaptations may offer avenues for engineering crop resilience. As discussed previously, several resurrection plants prime for a dehydration event by accumulating osmolytes in well-watered, non-stress conditions, which sensitive plants only accumulate in response to drought stress [112] and, in some cases, too late to prevent damage [73]. Key osmolytes include proline, glycine betaine, and sugars such as sucrose and raffinose [55]. These compounds accumulate as the cellular water potential decreases, helping to balance the osmotic pressure inside cells, slowing water loss to delay turgor loss; they also serve as osmoprotectants to protect cellular components from damage [86]. This raises the possibility that a limited but active accumulation of osmoprotectants in the hydrated state could both extend the window of growth (by increasing the time taken to reach the TLP) and offer osmoprotection once the TLP has been reached, which might also lower the lethal water potential threshold. Such a strategy must be finely tuned to ensure effectiveness without causing a yield penalty under non-stress conditions.

Both drought and desiccation impair photosynthesis, but whereas most plants can only protect the photosynthetic machinery for a limited time during severe water deficits before they are irreversibly damaged or dismantled during drought-induced senescence, homoiochlorophyllous desiccation-tolerant plants maintain the photosynthetic apparatus largely intact in the dried state [86]. As mentioned earlier, ELIP proteins appear to play a significant role in chloroplast protection during drying. ELIP proteins are thought to bind to chlorophyll and perhaps other components of the light-harvesting complex and protect them from photo-oxidative damage [113,114]. Transcripts of ELIP genes increase significantly in abundance in desiccation-tolerant plants under dehydration [93,115–117]. Overexpression of the C. *plantagineum* ELIP gene in *Medicago truncatula* improved the recovery from dehydration compared to wild-type plants [118]. The importance of ELIP proteins in VDT is highlighted by the observation that the genomes of resurrection plants exhibit a significant expansion of ELIP genes, which is not the case for desiccation-sensitive species. For instance, pea, tobacco, or tomato genomes contain only one or two ELIP genes, compared to 10–74 in the genome of resurrection plants [119–121]. This suggests that increasing the number of ELIP genes or their expression in crops might significantly improve chloroplast stability and enhance drought tolerance.

Another important component of VDT is the induction of LEA proteins during dehydration. These proteins serve multiple functions: membrane stabilization, preventing protein aggregation, and scavenging ROS. While the LEA subfamilies activated under drought stress are similar in desiccation-tolerant and sensitive plants, desiccation-tolerant species often display a broader and more dynamic expression of LEA genes. For example, the desiccation-tolerant plants *C. plantagineum, Oropetium thomaeum*, and *D. hygrometricum* show greater activation of LEA genes in response to dehydration than does *Arabidopsis* [92,122–124]. This robust molecular response regarding LEA gene expression resembles that seen during seed maturation, where LEA proteins play a key role in the desiccation phase of seed maturation. The broader and more dynamic expression of LEA genes in desiccation-tolerant plants hints at molecular flexibility, as seen in the protective mechanisms in seeds, enabling these plants to enter a reversible state of quiescence during extreme water stress. The difference in tolerance level to dehydration in a resurrection species versus a sensitive one might lie in the number of induced LEA proteins and the nature and duration of the response. Recently, LEA proteins have been shown to undergo a liquid–liquid phase separation and stabilize cellular structures by forming protein condensates [125]. This feature of LEA proteins makes them a potential target for improving the protection of critical cellular components during drought stress via genetic engineering.

Antioxidant enzyme activity in drought-tolerant plants increases incrementally as water availability declines but remains within a manageable threshold while the plant is still metabolically active [126,127]. The enzymes involved, SOD, CAT and ascorbate peroxidase (APX), work continuously to manage superoxide anions and hydrogen peroxide levels, two major ROS molecules [128]. Drought-tolerant plants primarily activate their antioxidant enzymes to prevent oxidative stress before it reaches damaging levels and maintain cellular integrity during moderate water stress [23]. The magnitude of the antioxidant response is significantly higher in desiccation-tolerant plants because extreme dehydration leads to higher levels of ROS and a severe oxidative burst upon rehydration [129,130]. Sister group contrasts using *Eragrostis* species demonstrated that antioxidant enzymatic activity halts below 50% relative water content (RWC) in desiccation-sensitive species, while the same enzymes remain active in the dried state (5% RWC) of the desiccation-tolerant grass *Eragrostis nindensis* [131]. It is improbable that these enzymes are active *in vivo* at this very low water content, but they likely remain functional to detoxify the rapid burst of ROS generated during recovery (rehydration). In addition, desiccation-tolerant plants accumulate high concentrations of secondary metabolites with antioxidant properties during dehydration. For example, about 70% of the dry mass of the desiccated leaves of the woody resurrection plant *Myrothamnus flabellifolia* is the potent phenolic antioxidant 3,4,5 tri-*O*-galloylquinic acid [132]. Thus, engineering more effective antioxidant systems in crops could help mitigate the damage caused by drought.

Aquaporins are critical for drought tolerance, as they regulate water transport. The overexpression of certain aquaporins in *Gossypium hirsutum*, such as GhPIP2;7 and GhTIP2;1, has been demonstrated to enhance water-use efficiency and drought tolerance [133]. Moreover, the aquaporin GoPIP1, found in *Galega orientalis*, was linked to drought stress tolerance, demonstrating the involvement of aquaporins in improving water homeostasis in unfavourable conditions [134]. Future VDT research could focus on identifying efficient aquaporins from resurrection plants, which may increase water retention capacity and drought recovery in crops.

Alongside the induction of protection mechanisms for acquiring VDT, resurrection plants must also exhibit cellular repair mechanisms upon rehydration [11]. Organisms that survive rapid drying rates (e.g., air dryness within an hour or faster) heavily rely on repair mechanisms, such as some algae and bryophytes [135]. Among cellular constituents, membranes are particularly prone to irreversible damage due to membrane fusion and the loss of cellular compartmentalization during excessive water loss [12]. Several fatty acid metabolism-related transcripts accumulate during rehydration of dried gametophytes of the desiccation-tolerant moss *S. caninervis,* suggesting they are involved in membrane repair processes [136]. Additionally, damage can also be caused by the rapid water influx into the dry cells. The moss *Racomitrium canescens* can recover up to 70% of its water content within the first minute of rehydration [137]. During rehydration, proteins related to the reconstruction of the cytoskeleton and chromatin architecture were synthesized, again suggestive of an active cellular repair mechanism. Future attempts to engineer crop stress tolerance might also consider inducing repair mechanisms to limit damage during moderate stress or improve the time it takes to recover from a water deficit event.

## 6. Current research insight: from resurrection plants to resilient crops

Several studies have highlighted genes and pathways in resurrection plants as potential targets for improving abiotic stress tolerance in plants. However, few studies have introduced genes from desiccation-tolerant plants into crops. For instance, transgenic rice plants overexpressing an osmotin (*TlOsm*) gene from the resurrection grass *Tripogon loliiformis,* exhibited increased tolerance to drought, cold, and salinity stress compared to wild-type rice plants [138]. The *TlOsm* gene was localized in the plasma membrane, and the transgenic lines maintained higher membrane integrity and water retention than wild-type rice under stress conditions.

Most plants exhibit DT in specific tissues or during specific life cycle phases, such as reproductive structures. Current knowledge suggests that resurrection plants may have evolved VDT by recruiting and ‘re-wiring’ pre-existing genes rather than acquiring new genes. The unique ability of resurrection plants to withstand extreme stress conditions may be associated with specific changes in gene regulation that activate a range of abiotic stress protection mechanisms in vegetative tissues. Consequently, engineering upstream regulators has been proposed as a more practical approach to activate sets of genes or pathways crucial for tolerance. Among these regulators, TFs of the bHLH, bZIP, DREB, Hsf, and WRKY families have been cloned from resurrection plants and expressed in model species like *A. thaliana* (table 1). The expression of these VDT regulators has generally enhanced abiotic stress tolerance, particularly in drought conditions, compared to wild-type plants. For example, overexpression of the DREB transcription factor BaDBL1 from the desiccation-tolerant moss *B. argenteum* significantly increased *Arabidopsis*’s osmotic and salt stress tolerance [101]. Importantly, this study also demonstrated the potential of the *BaDBL1* gene in regulating lignin biosynthesis, and as a result, the transgenic lines exhibited higher lignin content under osmotic stress, further enhancing their resilience.

**Table 1.** Heterologous expression of genes of resurrection plants that confer increased abiotic stress tolerance. Transcription factors are indicated in bold.

species source	gene	approach	phenotype	ref.
*D. hygrometricum*	**BhbZIP60**	expression driven by 35S in *Arabidopsis*	overexpression of BhbZIP60 increased drought tolerance. BhbZIP60 is subjected to mRNA-splicing for its translocation from the ER to the nucleus.	[139]
*D. hygrometricum*	*BhLEA1; BhLEA2*	expression driven by 35S in tobacco	overexpressing *BhLEA* genes exhibited enhanced tolerance to drought stress. Increased antioxidant enzyme activity and reduced membrane permeability under drought stress were also observed. Additionally, BhLEA overexpression can stabilize some photosynthesis-related proteins under drought stress.	[140]
*D. hygrometricum*	**BhHsf1**	expression driven by 35S in *Arabidopsis* and tobacco	overexpressing BhHsf1 confers increased thermotolerance to *Arabidopsis* and tobacco. *Penalty*: BhHsf1 overexpression resulted in dwarf phenotype and other pleiotropic effects. Expression analysis indicates that the mitotic cell cycle might be blocked in transgenic lines. Additionally, its overexpression impairs nuclear endoreduplication.	[141]
*D. hygrometricum*	*BhGolS1*	expression driven by 35S and RD29A in tobacco	heterologous expression of this galactinol synthase showed improved drought tolerance and higher survival after rewatering. Ectopic/drought-induced expression of BhGolS1 resulted in accumulation of galactinol and raffinose in transgenic plants.	[94]
*B. argenteum*	**BaDBL1**	expression driven by 35S in *Arabidopsis*	overexpression of DREB TF significantly increased osmotic and salt stress tolerance by decreasing ROS and increasing antioxidant enzyme activity. Its overexpression activates the lignin biosynthesis pathway under osmotic stress.	[101]
*C. plantagineum*	**CpMYB10**	expression driven by 35S in *Arabidopsis*	plants overexpressing CpMYB1 improved their drought and salt stress tolerance. Additionally, transgenic seedlings exhibited enhanced osmotic stress tolerance.	[95]
*M. flabellifolia*	**MfWRKY7,** **MfWRKY40,** **MfWRKY41,** **MfWRKY70**	expression driven by 35S in *Arabidopsis*	overexpression of these TFs enhanced drought and salt stress tolerance by promoting primary root growth and improving water retention capacity via narrowing stomata aperture, increased antioxidant enzyme activity and proline accumulation.	[94] [142] [143] [144]
*M. flabellifolia*	**MfbHLH38,** **MfbHLH145,** **MfPIF1**	expression driven by 35S in *Arabidopsis*	overexpression of these bHLH TFs enhanced tolerance to drought and salt stress by promoting root development (primary root growth; and number of lateral roots for MfbHLH145). Transgenic plants showed increased osmolyte accumulation and ROS-scavenging activity. Specifically, the overexpression of MfbHLH38 promoted ABA synthesis.	[145] [146] [147]
*T. loliiformis*	*TlOsm*	expression driven by Ubi in rice	plants overexpressing this osmotin gene exhibit enhanced tolerance to salinity, drought, and cold stress. *Penalty*: Under unstressed conditions, the growth of the TlOsm lines was reduced compared to wild-type plants.	[138]

Strategies to improve crop resilience to drought, such as engineering hormone signalling or targeting plant development, have been recently reviewed [44,148]. Resurrection plants represent promising genetic sources of novel genetic components that could enhance these targets for drought survival in crops. An example was the discovery of a MYB TF from the desiccation-tolerant moss *S. ruralis*, which acts as a negative regulator of the ABA-dependent stress response [149] .

Genomics has provided an unprecedented advance in identifying key genomic features for important agronomic traits [150]. Current technological sequencing innovations have facilitated genome reference generation for several desiccation-tolerant species [90–92,149,151–154]. Such genome references are crucial for dissecting and identifying the genetic basis for VDT. Resurrection plants as non-model organisms present difficulties and challenges due to their limited molecular toolkits, gene transfer protocols, and lack of resources. Therefore, a systems biology approach integrating multiple omics technologies is necessary to identify candidate regulators, gene sets, or pathways as targets for engineering stress tolerance in crops [83,155].

## 7. Exploring untapped research gaps and prospects

Most of what we know of how resurrection plants survive desiccation comes from studies focused on aerial tissues. However, some studies have shown differences in the desiccation response between shoots and roots within the same species [156]. Studies of the desiccation-tolerant dicot *Haberlea rhodopensis* demonstrated that its desiccation survival relies upon antioxidative defence mechanisms that protect its roots during drying and in the dried state [157]. Similarly, *C. plantagineum* exhibits tissue-specific responses in both roots and leaves during desiccation and recovery, resulting in distinct gene expression patterns for both organs [158]. Roots function as energy reservoirs during drought in these plants by storing carbohydrates crucial for survival. Studies on *T. loliiformis* have demonstrated that roots maintain higher sucrose and trehalose-6-phosphate (T6P) levels than shoots during dehydration, preventing autophagy. The metabolite T6P is a vital regulator of carbohydrate metabolism and contributes to drought resilience [159,160]. *C. plantagineum* accumulates high levels of sucrose and octulose in its roots, which act as osmoprotectants and stabilize cellular structures during desiccation [161]. Root respiration is typically downregulated during dehydration to conserve energy. For instance, root respiration rates in *H. rhodopensis* decrease during desiccation, conserving energy [162]. Beyond respiration, roots accumulate stress-related metabolites and proteins supporting DT. In *C. plantagineum*, metabolic shifts help roots survive dehydration and aid in the rapid regeneration of above-ground tissues once water becomes available again [161]. Their ability to modulate energy reserves, autophagy suppression, and tailored respiration responses highlights the unique strategies resurrection plants employ to endure and recover from desiccation, offering new genetic targets for crop improvement.

Roots also support plant–microbe interactions that are essential to plant fitness. Studies addressing the constituents and activity of the microbial communities associated with resurrection plants are limited despite growing evidence of the importance of plant microbiota in plant host performance. Tebele *et al.* [163] explored the microbiome of *M. flabellifolia* and uncovered its ability to aid in surviving drought conditions, providing insight into the interaction of the root-soil microbiome that enhances drought resilience. Since microbes can potentially improve resilience to numerous biotic and abiotic stresses [164–166], these interactions might play a prominent role in VDT and is another area of interest to explore. Additionally, root plasticity and hydraulic conductivity play a critical role in influencing water acquisition under changing soil moisture conditions [167]. Given the essential role of roots in plant health, future VDT research should further explore their tissue-specific responses and contributions to desiccation survival.

Resurrection plants suppress drought-induced senescence to survive extreme dehydration [12], opening the possibility for crop improvement, where ‘StayGreen’ phenotypes with delayed senescence have been linked to improved stress tolerance [168]. Griffiths *et al*. [18] highlighted that the signals triggering the onset of senescence are blocked in young tissues of resurrection plants but activated in older non-tolerant tissues [18]. Radermacher *et al*. [169] compared senescent and surviving tissues of *X. schlechteri* and observed that both initiated DT processes, but when the water level dropped to less than 35% RWC, age-related senescence was triggered in older tissues in a fashion similar to that seen for desiccation-sensitive plants, but not in younger tolerant tissues [169]. ABA and jasmonic acid hormones are critical in delaying senescence and regulating the response to dehydration [170]. There is still limited information on how desiccation-tolerant plants suppress drought-induced senescence. However, it has been proposed to be related to nitrogen mobilization and autophagy [12]. Still, this area of research needs attention to fully understand the mechanisms involved and their possible transfer to crops for improved drought tolerance.

The future direction in the field should focus on gaining a more profound knowledge of the complex regulatory responses activated in resurrection plants to establish the myriads of cellular protection mechanisms. The emergence of advanced technologies such as single-cell and spatial transcriptomics, along with high spatial resolution metabolomics, are revolutionizing plant research, but their use is still limited to laboratory models. Using such technologies in VDT studies could expand our understanding in unprecedented ways by unlocking the cell-specific responses to dehydration and identifying potential mobile signalling mechanisms that activate VDT in some plant species. Such knowledge could then be applied to develop more tolerant and robust crops to better adapt agricultural practices globally to this changing environment.

In summary, resurrection plants offer valuable insights into potential mechanisms for reducing crop yield losses during mild to moderate droughts, benefiting large-scale farmers with limited irrigation resources. More importantly, these insights could help develop crop varieties with enhanced tolerance to severe drought, which would be especially valuable for subsistence farmers in developing countries, where even small yields can significantly reduce the risk of famine.

## Data Availability

This article has no additional data.
